# Effects of highly active antiretroviral therapy on semen parameters of a cohort of 770 HIV-1 infected men

**DOI:** 10.1371/journal.pone.0212194

**Published:** 2019-02-21

**Authors:** Valeria Savasi, Francesca Parisi, Monica Oneta, Arianna Laoreti, Bina Parrilla, Piergiorgio Duca, Irene Cetin

**Affiliations:** 1 Unit of Obstetrics and Gynecology, Department of Biomedical and Clinical Sciences, ASST Fatebenefratelli Sacco, Hospital “L. Sacco”, University of Milan, Milan, Italy; 2 Centre for Fetal Research Giorgio Pardi, University of Milan, Milan, Italy; 3 Statistical and Biometry Unit, Department of Biomedical and Clinical Sciences, Hospital “L. Sacco”, University of Milan, Milan, Italy; CEA, FRANCE

## Abstract

**Background:**

HIV-1 infected patients show impaired semen parameters. Currently, it is not clear whether HIV-1 infection itself or antiretroviral therapy have an effect on semen parameters. We aim evaluate semen quality in a large cohort of fertile HIV-1 infected men under stable highly active antiretroviral therapy (HAART) and to assess the effect of HAART type and duration on semen parameters.

**Materials and methods:**

Between January 2010 and June 2014, we enrolled in a retrospective case-control study 770 HIV-1 patients under stable HAART asking a reproductive counselling with their HIV negative partner. Co-infections with HBV or HCV, genital tract infections and known causes of infertility represented exclusion criteria. Semen samples were analysed and compared with the WHO reference values. A multivariate analysis including HAART type and duration, age, viral load and CD4 count, was performed on 600 patients out of 770.

**Results:**

The median values of all semen parameters were significantly lower among HIV-1 infected patients compared to the WHO reference group, with a significant proportion of patients having values below the 5th percentile of the WHO reference value. In a multivariate analysis, only age and viral load negatively impacted progressive motility (β -0.3 (95% CI: -0.5; -0.0) %, p<0.05) and semen morphology (β -0.00 (95% CI: -0.00; -0.00) %, p≤0.01), while no associations were detected as regards HAART type and duration.

**Conclusions:**

HIV-1 infected patients showed a significant impairment of semen parameters compared to the reference values. HAART type and duration showed no associations with semen quality. Further research is needed to investigate implications for clinical care of HIV infected men desiring a child.

## Introduction

The likelihood of detecting HIV-RNA in semen of infected men has been shown to be extremely low in cases of prolonged efficient highly active antiretroviral therapy (HAART) [1–4]. For this reason natural conception may be considered as a safe option in HIV discordant couples, based on the very low probability of HIV sexual transmission. In this context, the assessment of fertility in HIV-infected men has gain increasing importance over the last years [5]. Several studies have reported semen alterations in HIV-infected men, such as decreased motility, sperm concentration, total sperm count and volume ejaculate [6–11]. Nevertheless, results are still controversial [12, 13]. Recently, Frapsauce et al. showed little or no influence of Nucleoside Reverse Transcriptase Inhibitors (NRTI), Protease Inhibitors (IP) and Nevirapine (NVP) on semen parameters [14]. By contrast, the exposure to efavirenz (EFV) has been associated with detrimental effects on semen parameters. Moreover, other studies have investigated the effect of HAART on sperm DNA integrity, showing mitochondrial damage, changes in spermatozoa metabolism, reduction of sperm motility and fertilization competence [11].

The aim of this study was to evaluate semen parameters of a large cohort of HIV-1 infected men under HAART compared to the World Health Organization (WHO) reference group and to assess the effect of HAART type and duration on semen parameters in a multivariate model, considering possible confounding factors, including age and viral load.

## Materials and methods

The protocol was approved by the Medical Ethical of our Institution (Comitato Etico area 1), ASST FBF Sacco—P.O. L. Sacco, University of Milan, and all participants signed a written informed consent and all patients signed a written informed consent before participation.

We designed a case-control study including semen samples from HIV-1 infected patients asking reproductive counselling with their HIV negative partner between January 2010 and June 2014 in our Assisted Reproduction Unit, Hospital “L. Sacco”, University of Milan, Italy. The protocol has been approved by the local medical ethics committee and all patients signed a written informed consent before participation.

### Study population

HIV-1 infected men requiring medical counselling on their risk of HIV transmission and available conception modes with their negative partner were eligible for inclusion. All included patients had never attempt spontaneous conception before the counselling and were thus considered a fertile population. All patients were at least 18 years old, under stable HAART and without any clinical signs of their disease. For all included patients, drugs abuse was stopped at least five years before inclusion and body mass index was between 18 and 25 kg/m^2^. Co-infections with HBV or HCV, previous diagnosis of infertility and any known cause of male infertility represented exclusion criteria, including varicocele, previous testicular surgery, endocrine dysfunction or cancer. Mycoplasma Genitalium, Chlamydia trachomatis and Neisseria Gonorrhoeae infections assessed by urethral swabs were additional exclusion criteria. In accordance to the Italian Guidelines for the management of the HIV infection, an annual dosage of vitamin D levels and supplementation in case of deficiency were performed. General questionnaires reporting items on demographic characteristics and medical history were collected. All available markers of HIV-1 disease, including CDC stage, current CD4+ T lymphocytes count, HIV-1 RNA in blood by RT-PCR and HAART duration and type, were recorded. HAART type groups were categorized as follows: 1) NRTI; 2) NRTI + IP; 3) NRTI + Non-nucleoside reverse transcriptase (NNRTI).

### Semen analysis

Semen samples were obtained for all patients by masturbation after 3–5 days of sexual abstinence. All samples were analysed by one biologist (O.M.) in the same laboratory within 2 hours from ejaculation according to the WHO recommendations [15] using manual microscopy. Motility was classified according to the WHO criteria as follows: a, rapid progressive spermatozoa; b, slow progressive spermatozoa; c, non-progressive spermatozoa; and d, immotile spermatozoa. The sperm concentration was calculated by a Makler chamber. The total sperm count was calculated as: total sperm count (x10^6^/mL) x ejaculate volume (in mL). The Papanicolaou smear for staining of spermatozoa was adopted for morphological evaluations. Semen samples with sexual abstinence >7 days, sperm concentration <1000000/mL, positive sperm culture, leukocyte count above the WHO threshold (>1 million/mL) and increased sperm viscosity were excluded from the final analysis.

### Statistical analyses

Appropriate descriptive statistics were calculated, including median and range for quantitative variables and absolute frequencies and percentages for categorical variables.

The Chi square test was applied to compare semen characteristics between the study population and the WHO 2010 reference values, classifying patients as below or above the lower fifth percentile. Additionally, baseline characteristics and semen parameters were compared across HAART type groups using the Kruskal-Wallis test. The same test was performed to compare HAART duration subgroups according to HAART type. Multivariable associations were assessed using a general linear model with HAART type and duration, age, viral load and CD4 count as dependent variables and semen parameters as independent variables. This model provides information about the strength of the relationship between the independent and dependent variables, independently on any interrelationship among the dependent variables, in contrast with the univariate analyses. Finally, a subgroup analysis on aviremic patients (<50 copies/ml, n = 413) was additionally performed.

Statistical analysis was performed using IBM-SPSS statistical package version 21. P-values ≤0.05 were considered significant.

## Results

**Fig 1:**
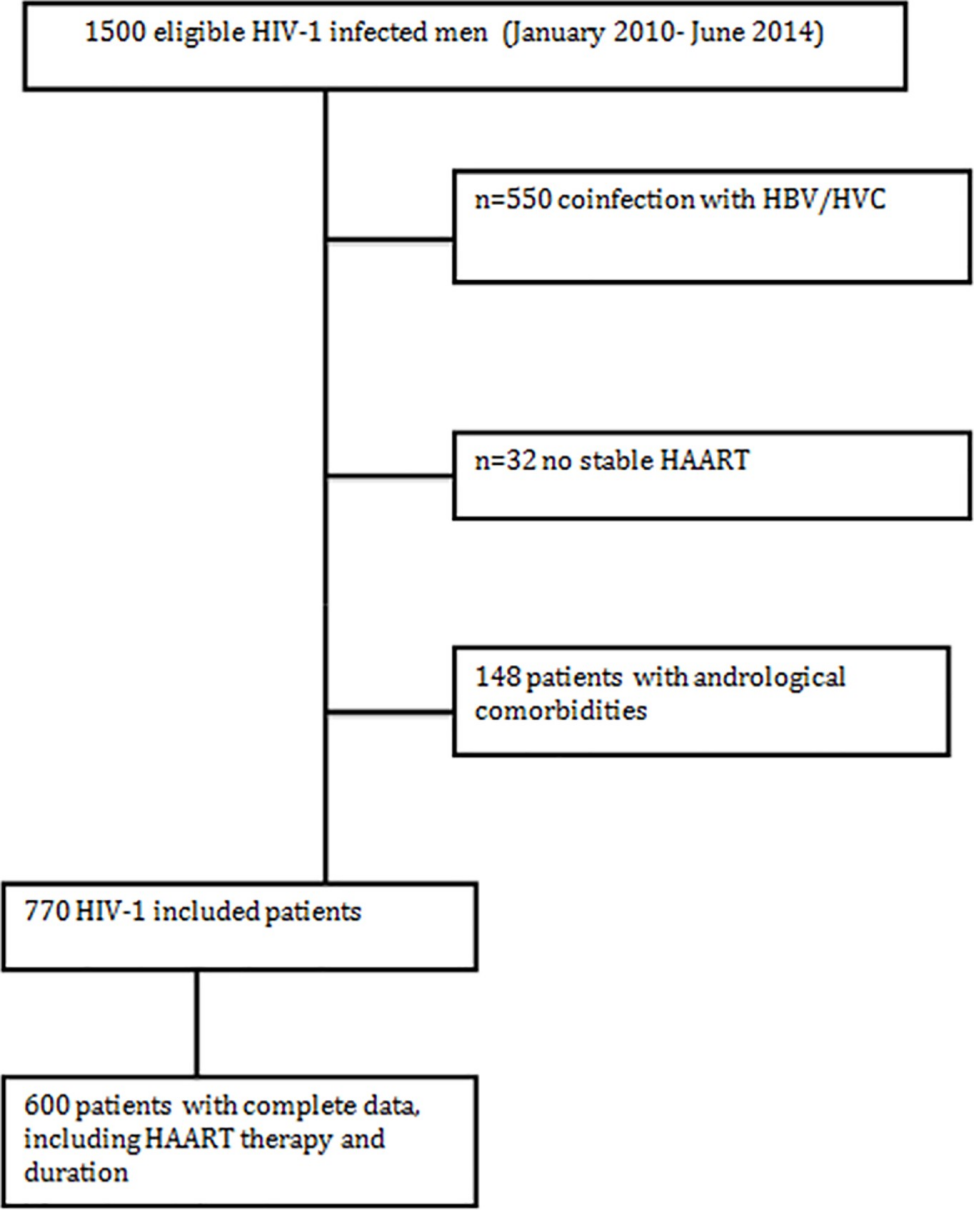
Flow chart of the study population.

770 HIV-1 infected patients were included in the analysis. Fig 1 shows the flow chart of the study population. As required by the study protocol, all patients were under stable HAART, but HAART type and duration were both known in a subgroup of 600 patients.

The route of HIV-1 transmission was sexual (52%), through intravenous drug use (38%) or blood product transfusion (2.2%), unknown (6.9%), professional (0.7%), and vertical (0.3%). Table 1 shows the baseline characteristics of the total study population according to HAART type.

**Table 1:** Baseline characteristics of the study population and HAART study groups.

Baseline characteristic	Study population n = 770	NRTI (n = 96)	NRTI+IP (n = 180)	NRTI+ NNRTI (n = 335)	p-value
Age (years), median (range)	40 (26–59)	40 (28–59)	40 (28–54)	40 (26–54)	0.86
Duration of HAART (years), median (range) *	11.67±7.43	9 (1–27)	11 (1–35)	12 (1–38)	0.14
**Viral load** (copies/mL)					
Detectable Undetectable	300 (39%) 470 (61%)	30%	38%	34%	0.18
CD4 count (cells/mm^3^)	555±272.1	460 (33–1573)	540 (149–1923)	503 (29–1670)	0.10

Baseline characteristics were compared across HAART type groups using Kruskal-Wallis test. HAART type and duration was unknown for 170 included patients. NRTI: reverse transcriptase inhibitor NNRTI: Non-nucleoside reverse transcriptase IP: protease inhibitor.

* HAART duration was recorded in a subgroup of 600 patients.

### Semen parameters of the study population

**Fig 2:**
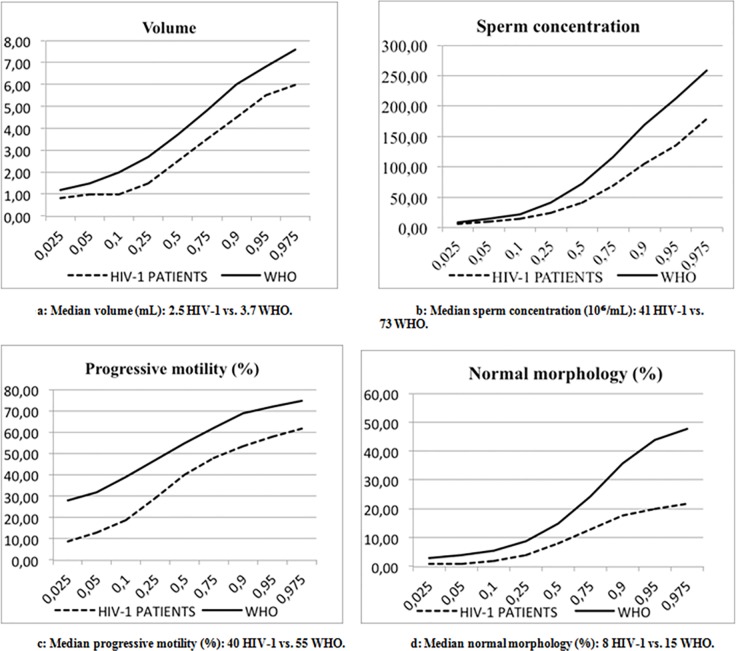
Distribution of semen parameters based on percentiles in the total study population and the WHO reference group. Semen parameters of HIV-1 infected men, including median volume (a), sperm concentration (b), progressive motility (c), normal morphology (d) and total sperm count were significantly decreased compared to the WHO distribution.

Table 2 shows semen parameters of the total study population compared to the WHO reference values. For each semen parameter, the percentage of patients with seminal values below the fifth percentile of WHO reference values was significantly increased. Fig 2 shows the distribution of semen parameters of the total study population and the WHO reference group, based on percentiles. Semen parameters of HIV-1 infected men, including median volume, sperm concentration, total sperm count, progressive motility and normal morphology, were significantly decreased compared to the WHO distribution.

**Table 2:** Comparisons between semen parameters of the total study population and the WHO 2010 reference values.

Semen parameters	Study population (n = 770)	WHO 5th percentile	Patients (%) outside WHO reference values	p-value
**Volume (ml)**	2.5 (0.2–9)	1.5	25.6	<0.05
**Sperm concentration (x10**^**6**^**/ml)**	41 (1.05–880)	15	12.2	<0.05
**Total sperm count (x10⁶)**	96.8 (0.26–1320)	39	15.3	<0.05
**Progressive motility** (**%)**	40 (0–95)	32	31.7	<0.05
**Normal morphology (%)**	8 (0–50)	4	28.2	<0.05

To assess the effect of HAART type on semen quality, firstly the non-parametric test showed comparable semen parameters among different groups of HAART type, with no significant differences for all the studied parameters (data not shown). Table 3 additionally shows the results from the general linear model performed in the subgroup of 600 patients out of 770 with known HAART type and duration. HAART type and duration were not associated with semen parameters, whereas only age and viral load significantly impacted progressive motility and morphology in a multivariate model.

**Table 3:** Results from the general linear model on semen parameters in a subgroup of 600 patients.

Dependent variable	Parameter estimates β (95% CI)	p-value
**Volume (ml)**		
**HAART type**	-0.85 (-0.43; 0.26)	0.63
**HAART duration**	-0.02 (-0.03; 0.00)	0.09
**Age**	-0.01 (-0.03; 0.02)	0.60
**Viral load**	-0.00 (-0.00; 0.00)	0.08
**CD4 count**	0.00 (-0.00; 0.00)	0.52
**Sperm concentration (x10**^**6**^**/ml)**		
**HAART type**	-10.5 (-24.6; 3.6)	0.14
**HAART duration**	0.3 (-0.4; 1.0)	0.42
**Age**	-0.4 (-1.5; 0.7)	0.48
**Viral load**	-0.0 (-0.0; 0.0)	0.11
**CD4 count**	-0.0 (-0.0; 0.0)	0.80
**Progressive motility (%)**		
**HAART type**	2.6 (-0.9; 6.1)	0.14
**HAART duration**	0.0 (-0.1; 0.2)	0.64
**Age**	**-0.3 (-0.5; -0.0)**	**0.03**
**Viral load**	-0.0 (-0.0; 0.0)	0.30
**CD4 count**	0.0 (-0.0; 0.0)	0.67
**Morphology (%)**		
**HAART type**	1.17 (-0.37; 2.71)	0.14
**HAART duration**	0.00 (-0.08; 0.08)	0.95
**Age**	-0.04 (-0.16; 0.08)	0.51
**Viral load**	**-0.00 (-0.00; -0.00)**	**0.01**
**CD4 count**	0.00 (-0.00; 0.00)	0.90

Values are regression coefficients (95% confidence interval) from the general linear model that reflect differences in semen parameters per standard-deviation scores change in age, HAART duration, viral load and CD4 count and per unit of change in HAART type (1: NRTI, 2: NRTI+IP, 3: NRTI+ NNRTI). The analysis was performed in the subgroup of 600 patients with known HAART type and duration.

The comparison of semen characteristics between the study population and the WHO 2010 reference group was performed using the Chi square test, classifying patients as below or above the lower fifth percentile. WHO: World Health Organization.

The subgroup analysis on aviremic patients (n = 413) showed no associations between HAART type and semen parameters, whereas a significant negative association was detected between HAART duration and semen volume (S1 Table). Finally, we found no significant differences in semen parameters between the subgroups of HAART duration in each HAART type group (S2 Table).

## Discussion

This study showed impaired volume, sperm concentration, total sperm count, progressive motility, and morphology, among otherwise fertile HIV-1 infected men under HAART compared to the WHO reference values. HAART type and duration did not affect semen quality in a multivariate model including age, viral load and CD4 count and in a well-defined HIV population. Our results strongly underline that HIV-1 infected men under therapy have impaired semen parameters irrespectively of HAART type and duration, as additionally confirmed by the analysis on subgroups of HAART duration. The same model, showed a negative association between HAART duration and semen volume in the subgroup of aviremic patients.

Previous studies compared semen parameters of HIV-1 infected men with various control groups [16, 6, 7, 8, 10]. We compared semen parameters of 770 HIV-1 infected men with the large dataset of the WHO reference values (more than 2000 individuals), as the most representative group of fertile men. A recent case–control study compared semen characteristics of HIV-1 infected patients receiving different antiretroviral regimens or never treated [14]. The authors reported that semen characteristics were similar between never treated patients and those receiving NRTIs alone or in combination with PI or NVP. In contrast, a marked drop (about 60%) of rapid spermatozoa percentage was found in the group of patients receiving an EFV-containing regimen, together with a significant decrease of total progressive motility (about 25%). This study shows a smaller sample size and does not take into account HAART duration. Despite a chronic therapy could theoretically impact semen quality, our and previous results do not support this hypothesis. Pilatz et al [17] compared semen parameters in a smaller sample of 116 HIV-1 positive patients under HAART to the WHO reference values and found that, in each category of semen parameters, about 25% of patients had semen parameters below the fifth percentile of WHO reference values, in line with our results. More recently, a prospective study conducted on 139 HIV-infected men showed median values of semen parameters within the normal WHO range, but up to 19% of HIV-positive males under stable therapy showed at least one parameter of semen quality below the normal range [18]. Morever, this study showed no associations between HAART duration and semen quality, in line with our findings [18]. Conversely, a significant association between exposure to EFV and the presence of sperm dysmotility was identified by Frapsauce et al. [14]. This was explained by the impaired sperm motility potentially resulting from flagellar dysfunction. The energy requirement for flagellar beating comes from the hydrolysis of ATP produced by the mitochondrial oxidative phosphorylation and glycolysis [19]. Since most HAART regimens include one NRTI or more, which has been associated with mitochondrial toxicity, a dysfunction of sperm mitochondrial respiration induced by NRTIs has been controversially proposed to explain the impaired motility observed in HIV-infected men [20–22]. In our cohort, all patients were under HAART with three different regimens, including EFV, and no associations were detected between HAART type and semen parameters in a multivariate analysis. Some authors proposed a possible association between exposure to EFV and impaired sperm motility due to vitamin D deficiency. A recent analysis from the Swiss HIV cohort study found a high prevalence of vitamin D deficiency in these patients, with respectively 42–52% of patients being deficient in spring and 14–18% in fall [23]. The authors stated that, in order to reach the target level of 75 nmol/l or higher for maximal vitamin D benefits, as many as 76–95% of the patients would be qualified for vitamin D supplementation. In accordance to this, the Italian Guidelines for the treatment and management of the HIV infection recommend an annual dosage of vitamin D levels and supplementation in case of deficiency. For this reason, our study population was screened and eventually supplemented for vitamin D deficiency, thus probably explaining our results of comparable motility according to the HAART type.

The mechanisms underlying the observed semen alterations in HIV-infected men remains unclear, although several hypotheses have been proposed. Men with advanced HIV infection, and particularly those with AIDS status, have abnormal sperm or abnormal spermatogenesis [24]. In this case, however, the severity of the disease could be the cause of the poor spermatogenesis. In HIV asymptomatic patients, a decreased ejaculate volume may be due to reduced accessory gland secretions or ejaculatory dysfunction. Dysfunction of the prostate and seminal vesicles, which are responsible for about 90–95% of the ejaculate volume, could be due to past or silent inflammation or infection, virus cell gland colonization or the effects of HAART drugs in the genital tract [25]. The virus could also increase reactive species of oxygen (ROS) production and decrease the presence of antioxidants [26, 27]. Furthermore, some authors found that infected macrophages can interact with the Leydig cells, leading to a reduction in free testosterone concentrations through the inhibition of steroidogenesis or dysfunction of the hypothalamus. This could lead to a degeneration of the seminal epithelium [24]. Our population included 38% of subjects infected through intravenous drug use. As possible confounding factors, coinfections and other chronic therapies represented exclusion criteria. On the other hand, it is important to know if substance abuse has an independent effect on semen quality. Unfortunately, there are not many data regarding this topic. Some authors reported an effect of heroin abuse on sperm maturity, motility and morphology, but only during the period of drug use [28]. In our study population, drug abuse was stopped at least five years before, thus reducing the bias related to possible drug effects.

This study has several strengths. This is the largest cohort of HIV patients at their first seminal control in a Reproductive Centre, seeking assistance for reproductive purpose rather than other health or HIV related problems. Moreover, other sexually transmitted diseases were excluded. This led to a large study population well defined and homogeneous, reducing possible bias associated with infertility and other chronic diseases. All semen samples were additionally collected and analysed in the same laboratory and by the same biologist. Also some limitations need to be addressed. Firstly, although more than two semen analyses were performed in most patients, only one semen sample per patient was analysed as a reliable picture of the baseline fertility status. Unexpectedly, a significant proportion of the study sample was viremic with a single blood detection (38%), but according to the Italian guidelines only 66 patients (8.6%) identified a virological failure of a stable HAART (viremia> 1000 copies/ml). Nevertheless, we additionally performed a subgroup analysis on aviremic patients (<50 copies/ml) in order to investigate associations in a more representative and purified study sample, confirming the absence of associations between HAART type and semen parameters. Conversely, this analysis showed a negative association between HAART duration and semen volume, as a potential adverse effect of the chronic therapy.

A second crucial limitation is that we compared HIV men under stable therapy with the reference WHO values of fertile men. Ideally, the control group should be represented by fertile HIV infected untreated men, which represent a difficult cohort as all HIV patients requiring reproductive assistance are generally already under treatment. A third limitation is that the studied therapeutic regimens are not completely up to date, including in fact old combinations like three NRTIs. Longitudinal changes of the therapy were not subsequently recorded, although we collected HAART duration up to the recruitment. Moreover, new regimens, including integrase inhibitors, were not included in the analysis in order to have a more homogeneous study population, being represented only in few patients. The ideal control group should be represented by naïve HIV infected patients not under treatment, which is a difficult group to detect as most patients are usually addressed to our clinic by the infectivologist.

Finally, other confounding factors including smoking habit, alcohol use and duration of HIV infection were not available for the analysis.

Our study provides evidence of impaired conventional semen parameters of HIV-1 infected patients under stable HAART and therefore a possible altered fertility status. In addition, our results suggest that HAART type does not impact semen quality, as opposed to age and viral load. Currently, data regarding the semen quality in HIV infected patients still represent an uncertain and contradictory issue. However, since natural conception has been recently advocated as a safe option in HIV discordant couples desiring a child, it is mandatory for clinicians to understand whether HAART or HIV infection itself could have a detrimental effect on infected men’s fertility. According to our results, all serodiscordant couples with HIV infected men on HAART should be advised about the potential impairment of semen parameters and consider an early semen evaluation, in order to guide them through the attempt of natural conception or through assisted reproduction. No preferential therapy needs to be suggested in order to increase the reproductive potential.

## Supporting information

S1 Table Results from the general linear model on semen parameters in a subgroup of 413 aviremic patients. Values are regression coefficients (95% confidence interval) from the general linear model that reflect differences in semen parameters per standard-deviation scores change in age, HAART duration, viral load and CD4 count and per unit of change in HAART type (1: NRTI, 2: NRTI+IP, 3: NRTI+ NNRTI). The analysis was performed in the subgroup of 413 aviremic patients (< 50 copies /ml) with known HAART characteristics.
(DOCX)

S2 Table Semen parameters among subgroups of HAART type according to HAART duration. HAART type was defined as: 1, NRTI; 2: NRTI+IP; 3: NRTI+ NNRTI. HAART duration subgroups were defined according to the median duration for each HAART type group. The comparison between HAART duration subgroups was performed using Kruskall-Wallis test. N.s.: not significant.
(DOCX)
