# Supplementary material for: Effects of highly active antiretroviral therapy on semen parameters of a cohort of 770 HIV-1 infected men
Source: PLoS One. 2019 Feb 21;14(2):e0212194. doi: 10.1371/journal.pone.0212194 (PMC6383866; doi:10.1371/journal.pone.0212194)
Supplement: S1 Table — Values are regression coefficients (95% confidence interval) from the general linear model that reflect differences in semen parameters per standard-deviation scores change in age, HAART duration, viral load and CD4 count and per unit of change in HAART type (1: NRTI, 2: NRTI+IP, 3: NRTI+ NNRTI). The analysis was performed in the subgroup of 413 aviremic patients (< 50 copies /ml) with known HAART characteristics. (DOCX) [file pone.0212194.s001.docx]

**S Table 1. Results from the general linear model on semen parameters in a subgroup of 413 aviremic patients.**

| **Dependent variable** | **Parameter estimates**  β (95% CI) | **p-value** |
| --- | --- | --- |
| **Volume (ml)** | | |
| **HAART type** | -0.11 (-0.51; 0.28) | 0.58 |
| **HAART duration** | -0.02 (-0.04; 0.00) | **0.03** |
| **Age** | -0.00 (-0.03; 0.03) | 0.84 |
| **CD4 count** | 0.00 (-0.00; 0.00) | 0.30 |
| **Sperm concentration (x10^6^/ml)** | | |
| **HAART type** | -6.04 (-22.2; 10.1) | 0.46 |
| **HAART duration** | -0.11 (-0.96; 0.73) | 0.80 |
| **Age** | -0.48 (-0.70; 1.67) | 0.42 |
| **CD4 count** | -0.01 (-0.03; 0.01) | 0.57 |
| **Progressive motility (%)** | | |
| **HAART type** | 2.4 (-1.62; 6.32) | 0.25 |
| **HAART duration** | 0.00 (-0.20; 0.21) | 0.99 |
| **Age** | -0.25 (-0.54; -0.05) | 0.10 |
| **CD4 count** | 0.00(-0.00; 0.00) | 0.40 |
| **Morphology (%)** | | |
| **HAART type** | 1.36 (-0.37; 3.08) | 0.12 |
| **HAART duration** | 0.02 (-0.11; 0.07) | 0.63 |
| **Age** | -0.01 (-0.14; 0.12) | 0.87 |
| **CD4 count** | -0.00 (-0.00; 0.00) | 0.21 |

Values are regression coefficients (95% confidence interval) from the general linear model that reflect differences in semen parameters per standard-deviation scores change in age, HAART duration, viral load and CD4 count and per unit of change in HAART type (1: NRTI, 2: NRTI+IP, 3: NRTI+ NNRTI). The analysis was performed in the subgroup of 413 aviremic patients (< 50 copies /ml) with known HAART characteristics.
