# Supplementary material for: Effects of highly active antiretroviral therapy on semen parameters of a cohort of 770 HIV-1 infected men
Source: PLoS One. 2019 Feb 21;14(2):e0212194. doi: 10.1371/journal.pone.0212194 (PMC6383866; doi:10.1371/journal.pone.0212194)
Supplement: S2 Table — HAART type was defined as: 1, NRTI; 2: NRTI+IP; 3: NRTI+ NNRTI. HAART duration subgroups were defined according to the median duration for each HAART type group. The comparison between HAART duration subgroups was performed using Kruskall-Wallis test. N.s.: not significant. (DOCX) [file pone.0212194.s002.docx]

**S Table 2. Semen parameters among subgroups of HAART type according to HAART duration.**

| **HAART type** | **HAART duration** | **Volume (ml),** median (range) | **Sperm concentration** (x10^6^ /ml)  median (range) | **Morphology (%)**  median (range) | **Motility (%)**  median (range) | **p** |
| --- | --- | --- | --- | --- | --- | --- |
| 1 | ≤9, n=47 | 2.5 (3.0-6.0) | 34 (8-140) | 11 (2-23) | 40 (16-95) | n.s. |
|  | >9, n=45 | 2.5 (0.5-6.0) | 43 (0-114) | 9 (1-20) | 41 (12-80) |  |
| 2 | ≤11, n=93 | 2.5 (0.7-6) | 52 (2-324) | 9 (1-25) | 40 (0-95) | n.s. |
|  | >11, n=84 | 2.4 (0.5-9.0) | 43 (4-230) | 8 (1-22) | 41.5 (2-68) |  |
| 3 | ≤12, n=172 | 2.5 (0.3-7.5) | 38 (1.4-880) | 7 (0-50) | 40 (1-74) | n.s. |
|  | >12, n=158 | 2.5 (0.4-7.0) | 47 (0.9-330) | 8 (1-27) | 40 (3-79) |  |

HAART type was defined as: 1, NRTI; 2: NRTI+IP; 3: NRTI+ NNRTI. HAART duration subgroups were defined according to the median duration for each HAART type group. The comparison between HAART duration subgroups was performed using Kruskall-Wallis test. N.s.: not significant.
